# Predictors of neurological outcome after out-of-hospital cardiac arrest: sex-based analysis: do males derive greater benefit from hypothermia management than females?

**DOI:** 10.1186/s12245-022-00447-z

**Published:** 2022-09-05

**Authors:** Emad M. Awad, Karin H. Humphries, Brian E. Grunau, Colleen M. Norris, Jim M. Christenson

**Affiliations:** 1 Faculty of Medicine, Experimental Medicine, University of British Columbia, 2775 Laurel Street, 10th Floor, Room 10117, Vancouver, BC V5Z 1M9 Canada; 2 BC RESURECT: BC Resuscitation Research Collaborative, Vancouver, British Columbia Canada; 3 Division of Cardiology, Faculty of Medicine, University of British Columbia, Vancouver, British Columbia Canada; 4 BC Centre for Improved Cardiovascular Health, Vancouver, British Columbia Canada; 5 Department of Emergency Medicine, St. Paul’s Hospital, Vancouver, British Columbia Canada; 6 Department of Emergency Medicine, University of British Columbia, Vancouver, British Columbia Canada; 7 Faculties of Nursing, Medicine, and School of Public Health, University of Alberta, Edmonton, Alberta Canada

**Keywords:** Resuscitation, Cardiac arrest, Sex differences, Neurological outcome

## Abstract

**Background:**

Previous studies of the effect of sex on after out-of-hospital cardiac arrest (OHCA) outcomes focused on survival to hospital discharge and 1-month survival. Studies on the effect of sex on neurological function after OHCA are still limited. The objective of this study was to identify the predictors of favorable neurological outcome and to examine the association between sex as a biological variable and favorable neurological outcome OHCA.

**Methods:**

Retrospective analyses of clustered data from the Resuscitation Outcomes Consortium multi-center randomized controlled trial (2011–2015). We included adults with non-traumatic OHCA and EMS-attended OHCA. We used multilevel logistic regression to examine the association between sex and favorable neurological outcomes (modified Rankin Scale) and to identify the predictors of favorable neurological outcome.

**Results:**

In total, 22,416 patients were included. Of those, 8109 (36.2%) were females. The multilevel analysis identified the following variables as significant predictors of favorable neurological outcome: younger age, shorter duration of EMS arrival to the scene, arrest in public location, witnessed arrest, bystander CPR, chest compression rate (CCR) of 100–120 compressions per minute, induction of hypothermia, and initial shockable rhythm. Two variables, insertion of an advanced airway and administration of epinephrine, were associated with poor neurological outcome. Our analysis showed that males have higher crude rates of survival with favorable neurological outcome (8.6 vs. 4.9%, *p* < 0.001). However, the adjusted rate was not significant. Further analyses showed that hypothermia had a significantly greater effect on males than females.

**Conclusions:**

Males had significantly higher crude rates of survival with favorable neurological outcome. However, the adjusted rate was not statistically significant. Males derived significantly greater benefit from hypothermia management than females, but this can possibly be explained by differences in arrest characteristics or in-hospital treatment. In-depth confirmatory studies on the hypothermia effect size by sex are required.

## Introduction

Sudden out-of-hospital cardiac arrest (OHCA) is a serious medical emergency affecting more than 350,000 North Americans yearly [1, 2]. Providing patients suffering from OHCA with optimal prehospital intervention improves the probability of return of spontaneous circulation (ROSC) [3–9]. Even OHCA patients who receive prehospital resuscitation and achieve ROSC are at risk of developing anoxic brain complications, including coma and death [10].

Many studies examined the predictors of survival with good neurological outcomes and identified the contributors to favorable neurological outcome as: younger age, shorter time to ROSC, witnessed arrest, initial rhythm being a shockable rhythm, and arrest due to cardiac etiology [9, 11–13]. In addition to prehospital treatment-related predictors, there is evidence suggesting that sex may influence survival. Previous studies of the effect of sex on OHCA outcomes focused on survival to hospital discharge [14–18] and 1-month survival [16, 19, 20]. Some other studies assessed the effect of sex on OHCA neurological outcome; however, they reported contradictory results. While some reported no significant difference by sex [15, 21], others reported male sex is associated with a favorable neurological function [22, 23]. In contrast, a recent study reported neurological outcome advantage in females [24]. The primary objective of this study was to assess whether sex as a biological variable has any association with favorable neurological outcome after OHCA. The secondary objective was to identify the predictors of favorable neurological outcome.

## Methods

### Design and setting

We analyzed data from the Resuscitation Outcome Consortium (ROC) Continuous Chest Compression (CCC) trial (June 2011 to May 2015) [25]. The ROC is a resuscitation research network with 10 clinical regions in the USA and Canada [26]. The ROC CCC trial was a cluster-randomized, trial that included 114 emergency medical service (EMS) agencies across eight sites in the USA and Canada. The participating EMS agencies were grouped into 47 clusters. The CCC trial included adults with non-traumatic OHCA who received chest compressions provided by EMS and excluded OHCA witnessed by EMS, patients pronounced dead on EMS arrival, and those with “do not resuscitate” orders. The trial examined the effect of chest compressions provided continuously (CCC) versus chest compressions interrupted for ventilation, on survival [25]. The trial results showed no significant difference in survival or neurological function between the continuous versus interrupted chest compression groups. A detailed report of the CCC trial has been published [25].

### Study population

From the CCC database, we created an analytic dataset that included males and females 18 years and older, with non-traumatic, EMS-attended OHCA. Cases with non-cardiac etiology of arrest and cases missing data on any of the key variables were excluded.

### Key variables of interest and measurements

The outcome variable of interest was neurological function at hospital discharge measured using a modified Rankin Scale (mRS), a scale from zero to six, where a scale of zero represents no neurological deficit and a scale of six means death [27]. For this study, a mRS of >3 was coded as (0) and indicated unfavorable neurological function, and a mRS of ≤ 3 was coded as [1] and indicated favorable neurologic function.

In addition to “sex,” a total of 10 independent variables known to be associated with short-term survival were screened as possible predictors for the neurological outcome. These included the following: age (per year), the interval from the 9-1-1 call to first to EMS arrival to the scene (per minute), location of arrest (public vs. private), bystander witnessed status (witnessed vs. unwitnessed), bystander cardiopulmonary resuscitation (CPR) (yes vs. no), chest compression rate (CCR), initial cardiac arrest rhythm (shockable vs. nonshockable), advanced airway placement (yes vs. no), administration of epinephrine (administered vs. not administered), and induction of hypothermia. Patients who had in-hospital cooling or continued hypothermia for prehospital at temp of 32 to 35 °C were coded as “yes.”

### Statistical analysis

We calculated descriptive statistics for baseline characteristics for the full cohort and stratified by sex. Continuous variables were presented as medians and interquartile ranges, as they were not normally distributed. Categorical variables were presented as counts and percentages. To explore associations between the potential predictors including “sex” and neurological outcome, we performed bivariate analyses using chi-square test for the categorical variables and point biserial correlation for the continuous variable. Bivariate relationships were assessed at a 5% level of significance.

To further examine the effect of sex and identify the significant prehospital predictors of favorable neurological outcome, we employed multilevel (hierarchical) logistic regression. Multilevel modeling was necessary to account for the nesting of patients within 47 clusters [28]. We ran the hierarchal logistic regression analyses as follows: first, we built an intercept-only model (null model) and estimated the intercept (𝜸𝟎𝟎) and variance (τ^2^). 𝜸𝟎𝟎 represents the average log odds of survival with favorable neurological outcome across all clusters, and *τ*^2^ represents the variance of clusters’ average log odds of survival with favorable neurological outcome [29]. Second, we calculated the intraclass correlation coefficient (ICC) and the design effect (DE). A DE value greater than two was considered an indication of the need for multilevel analysis [28]. Third, using a forward and backward variable selection technique, we built a series of multivariable mixed models (random intercept models with patient-level predictors). This allowed us to account for the clustering effect (patients nested within clusters). In these models, we included patient-level variables known to be associated with survival [30]. The patient-level variables were modeled as fixed effects, and the cluster was modeled as a random effect. The dependent variable was neurological function at hospital discharge (favorable vs. unfavorable). Fourth, to further examine the effect of sex, we stratified the sample by sex and ran the models in each of the sex subgroups. We then compared the predictors in each model for both the significance and effect sizes. Fifth, we examined for possible interactions between sex and other variables. We used the Akaike information criterion (AIC) to evaluate the overall fit of the models [31]. All analyses were performed using R version 4.0.1, Vienna, Austria. Ethics approval for this study was obtained from the University of British Columbia - Providence Health Care Research Ethics Board.

## Results

### Patients’ characteristics and univariate analyses

**Fig. 1:**
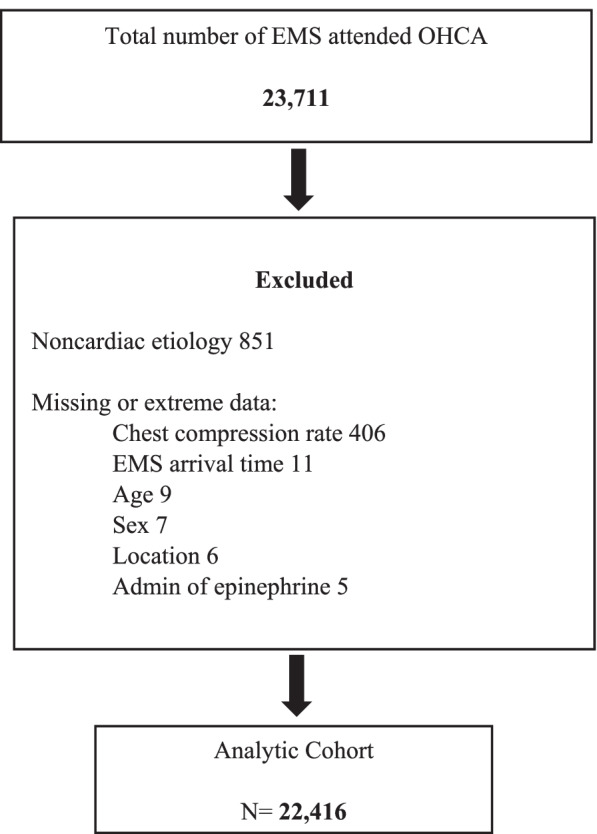
Study flow diagram

A total of 23,711 OHCA cases were included in the CCC trial database. After excluding patients who did not meet the inclusion criteria and those with missing data on any of the key variables, 22,416 patients were eligible and included in the analysis (Fig. 1). Of those, 8109 (36.2%) were females, 3249 (14.5%) arrested in a public location, 10,403 (46.4%) received bystander CPR, and 5187 (23.1%) had an initial shockable rhythm. The summary statistics for the study variables and the bivariate analyses results are shown in Table 1. When comparing the females to the males, the unadjusted analyses showed that females had a lower proportion of OHCA in public locations (10.1% vs. 17.0%, *p* < 0.001), a lower proportion with an initial shockable rhythm (14.1% vs. 28.3%, *p* < 0.001), and a lower proportion received hypothermia intervention (13.3% vs. 17.0%, *p* < 0.001). Among the full cohort, survival to hospital discharge was 8.7%, and survival with good neurological function was 7.3%. Males have higher crude rates of survival to hospital discharge than females (10.2% vs. 6.0%, *p* < 0.001) and survival with favorable neurological outcome (8.6 vs. 4.9%, *p* < 0.001) (Table 2).

**Table 1:** Patients’ characteristics and univariate associations with favorable neurological outcome

Study variable	***N*** = 22,416	Favorable neurological outcome	***P*** value
**EMS arrival interval (min)**	Median = 5.5 (4.2–6.9)	*r*_pb =_ 0.05^a^	< 0.01
**Age (years)**	Median = 69 (57–81)	*r*_pb =_ 0.13^b^	< 0.01
**Sex**			
Female	8109 (36.2%)	395 (4.9%)	< 0.001
Male	14,307 (63.8%)	1234 (8.6%)	
**Location of arrest**			
Non-public	19,167 (85.5%)	946 (4.9%)	< 0.001
Public	3249 (14.5%)	683 (21.0%)	
**Bystander witnessed**			
Unwitnessed	12,845 (57.3%)	325 (2.5%)	< 0.001
Witnessed	9571 (42.7%)	1304 (13.6%)	
**Bystander CPR**			
No	12,013 (53.6%)	568 (4.7%)	< 0.001
Yes	10,403 (46.4%)	1061 (10.2%)	
**Initial rhythm**			
Nonshockable	17,229 (76.9%)	328 (1.9%)	< 0.001
Shockable	5187 (23.1%)	1301 (25.1%)	
**Chest compression rate per min**			
50–99	3599 (16.1%)	196 (5.4%)	
100–120	17,705 (78.9%)	1370 (7.7%)	< 0.001
> 120	1112 (5.0%)	63 (5.7%)	
**Advanced airway**			
No	4958 (22.1%)	597 (36.6%)	< 0.001
Yes	17,458 (77.9%)	1032 (5.9%)	
**Admin of Epinephrine**			
No	3950 (17.6%)	899 (22.8%)	< 0.001
Yes	18,466 (82.4%)	730 (4.0%)	
**Hypothermia management**			
No	18,906 (84.3%)	629 (3.3%)	< 0.001
Yes	3510 (15.7%)	1000 (28.5%)	

^a,b^Point biserial correlation coefficient

**Table 2:** Sex differences in baseline characteristic, interventions, and outcome

Study variables	Total 22.416	Females 8109 (36.2%)	Males 14,307 (63.8%)	***P value***
**EMS arrival time, median (IQR)**	5.5 (4.2–6.9)	5.4 (4.1–6.7)	5.6 (5.3–6.9)	0.08
**Age, median (IQR)**	69 (57–81)	71 (57–82)	68 (55–79)	<0.001
**Arrest location**				
Nonpublic	19,167 (85.8%)	7293 (89.9%)	11,874 (83.0%)	<0.001
Public	3249 (14.2%)	816 (10.1%)	2433 (17.0%)	
**Bystander witness**				
Unwitnessed	12,845 (57.3%)	4946 (61.0%)	7899 (55.2%)	0.001
Witnessed	9571 (42.7%)	3163 (39.0%)	6408 (44.8%)	
**Bystander CPR**				
No	12,013 (53.6%)	4688 (57.8%)	7325 (51.2%)	< 0.001
Yes	10,403 (46.4%)	3421 (42.2%)	6982 (48.8%)	
**Initial rhythm**				
Nonshockable	17,229 (76.9%)	6966 (85.9%)	10,263 (71.7%)	< 0.001
Shockable	5187 (23.1%)	1143 (14.1%)	4044 (28.3%)	
**Advanced airway**				
No	4958 (22.1%)	1810 (22.3%)	3148 (22.0%)	0.80
Yes	17,458 (77.9%)	6299 (77.7%)	11,159 (78.0%)	
**Administration of epinephrine**				
No	3950 (17.6%)	1546 (19.1%)	2404 (16.8%)	
Yes	18,466 (82.4%)	6563 (80.9%)	11,903 (83.2%)	0.03
**Hypothermia management**				
No	18,906 (84.3%)	7031 (86.7%)	11,875 (83.0%)	
Yes	3510 (15.7%)	1078 (13.3%)	2432 (17.0%)	< 0.001
**Survival outcomes**				
**Hospital discharge**				
No	20,471 (91.3%)	7623 (94.0%)	12,848 (89.8%)	< 0.001
Yes	1945 (8.7%)	486 (6.0%)	1459 (10.2%)	
**Neurological outcomes**				
Unfavorable	20,778 (92.7%)	7714 (95.1%)	13,064 (91.4%)	< 0.001
Favorable	1638 (7.3%)	395 (4.9%)	1243 (8.6%)	

### Multivariable logistic regression analyses

#### Null model

In the intercept-only model (null model), the estimated intercept **γ00** was − 2.65, while the estimated variance of the random effects 𝝉^𝟐^ was 0.25 (95% CI 0.15–0.40, *p* < 0.001). This indicates that there was significant variability in the intercept among clusters. To allow further assessment for the clustering effect, we computed the ICC:

$$\mathrm{ICC}=\frac{\uptau^2}{\uptau^2+\frac{\pi^2}{3}}$$ where π = 3.14 [32]

$${\displaystyle \begin{array}{c}\mathrm{ICC}=\frac{0.25}{0.247+\left(3.14\ x\ 3.14\right)/3\ }\\ {}\mathrm{ICC}=\frac{0.25}{0.25+3.28\ }\\ {}\begin{array}{c}\mathrm{ICC}=0.07\\ {}\mathrm{DE}=1+\mathrm{ICC}\ \left(n-1\right),n=\mathrm{the}\ \mathrm{average}\ \mathrm{number}\ \mathrm{of}\ \mathrm{patients}\ \mathrm{per}\ \mathrm{cluster}.\\ {}\begin{array}{c}\mathrm{DE}=1+0.07\ \left(476-1\right)\\ {}\mathrm{DE}=34.25\end{array}\end{array}\end{array}}$$

ICC and DE values suggested that multilevel analysis is needed.

#### Random intercept with level one predictors model

In the series of models produced by the forward and backward variable selection procedure, sex remained statistically significant in favor of males. Only after adding two variables (hypothermia and initial rhythm) was sex no longer significant with *p* value slightly above the significance level (OR M vs. F 1.16, 95% CI 0.99–1.36, *p* = 0.06). The effects of all other potential predictors remained significant (Table 3).

**Table 3:** Hierarchical multivariable regression model for favorable neurological outcome

Variable	Full cohort ***N*** = 22,416		
	OR	(95% CI)	***P*** value
**EMS arrival interval (per min)**	0.92	0.89–0.94	< 0.001
**Age (per year)**	0.97	0.96–0.97	< 0.001
**Sex**			
Female	Ref		
Male	1.16	0.99–1.36	0.06
**Location of arrest**			
Not public	Ref		
Public	1.77	1.53–2.06	< 0.001
**Witness status**			
Unwitnessed	Ref		
Witnessed	3.02	2.76–3.55	< 0.001
**CPR status**			
No CPR	Ref		
Bystander CPR	1.29	1.12–1.49	< 0.001
**Chest compression rate**			
50–99	Ref		
100–120	1.59	1.31–1.95	< 0.001
> 120	0.97	0.65–1.44	0.86
**Initial cardiac rhythm**			
Nonshockable	Ref		
Shockable	6.48	5.40–7.75	< 0.001
**Epinephrine**			
No	Ref		
Yes	0.10	0.08–0.12	< 0.001
**Advanced airway**			
No	Ref		
Yes	0.59	0.49–0.69	< 0.001
**Hypothermia management**			
No	Ref		
Yes	5.03	4.07–6.15	< 0.001

AIC 161979.14

To further examine the effect of sex, we stratified the sample by sex and ran the models in each of the sex subgroups. We then compared the predictors in each model for both the significance and effect sizes. The significance and effect sizes were very similar in the male and female models except in the effect of hypothermia and initial rhythm. Hypothermia and initial rhythm had larger effect sizes in males than females (Table 4). This suggests that the effect of hypothermia and initial rhythms depend on the levels of “sex” variable (effects modification by sex). We thus ran three new models: 1—all predictors and the initial shockable rhythm by sex interaction, 2—all predictors, the initial rhythm by sex interaction, and hypothermia management by sex interaction, and 3—all predictors and the hypothermia management by sex interaction only (Table 5). The results of these analyses showed that the initial shockable rhythm by sex interaction was significant in favor of males. However, after adding the hypothermia by sex interaction, the initial rhythm by sex was no longer significant. Moreover, after removing initial rhythm by sex and including hypothermia by sex interaction only, the hypothermia by sex remained significant, i.e., hypothermia was significantly more effective in males than females, holding all other predictors constant (Table 5). The AIC showed that the last model that include all predictors and hypothermia by sex interaction was the best-fit model and therefore was accepted as the final model (Table 6). In this final model, the following variables were found to have a statistically significant positive impact on good neurological outcome: younger age, shorter duration of EMS arrival to the scene, arrest in public location, witnessed arrest, bystander CPR, CCR of 100–120 compressions per minute, initial shockable rhythm, and hypothermia by sex interaction.

**Table 4:** Hierarchical multivariable logistic regression analysis stratified by sex

Variable	Males model ***n*** = 14,307 (63.8%)			Females model ***n*** = 8109 (36.2%)		
	OR	(95% CI)	***P*** value	OR	(95% CI)	***P*** value
**EMS arrival (per min)**	0.91	0.88–0.94	< 0.001	0.96	0.94–0.98	< 0.001
**Age (per year)**	0.97	0.96–0.98	< 0.001	0.98	0.97–0.99	< 0.001
**Location of arrest**						
Not public						
Public	1.70	1.35–2.26	< 0.001	1.74	1.34–2.25	< 0.001
**Witness status**						
Unwitnessed				2.04	1.76–2.35	< 0.001
Bystander witnessed	2.90	2.40–3.51	< 0.001			
**CPR status**						
No CPR					1.03–1.49	0.006
Bystander CPR	1.35	1.15–1.60	< 0.001	1.24		
**Chest compression rate**						
**50–99**						
**100–120**	1.72	1.35–2.18	< 0.001	1.42	1.05–1.88	< 0.001
**>120**	1.01	0.63–1.61	0.96	0.95	0.57–1.56	0.84
**Initial rhythm**						
Nonshockable						< 0.001
Shockable	7.29	6.03–8.81	< 0.001	3.76	3.06–4.62	
**Epinephrine**						
No				0.27	0.21–0.33	< 0.001
Yes	0.09	0.08–0.11	< 0.001			
**Advanced airway**						
No						
Yes	0.55	0.44–0.68	< 0.001	0.45	0.38–0.52	< 0.001
**Hypothermia management**						
No				2.46	1.98–3.05	< 0.001
Yes	7.21	5.91–8.75	< 0.001

**Table 5:** Hierarchical multivariable regression models for favorable neurological outcome showing the interactions results

Model	OR	(95% CI)	***P*** value	AIC
**Model 1** All predictors + one interaction term (sex* initial shockable rhythm)	Sex * initial rhythm 1.44	1.35–1.54	0.001	161,569.65
**Model 2** All predictors + two interaction terms sex*initial shockable rhythm and sex*hypothermia	Sex * initial rhythm 0.81	0.59–1.13	0.221	161,617.85
	Sex * hypothermia 1.69	1.23–2.33	0.001	
**Model 3** All predictors + one interaction term (sex*hypothermia)	Sex * hypothermia 1.61	1.18–2.19	0.003	161,549.14

**Table 6:** Hierarchical multivariable regression model for favorable neurological outcome. Included one interaction (sex*hypothermia)

Variable	Full cohort N = 22,416		
	OR	(95% CI)	***P*** value
**EMS arrival interval (per min)**	0.92	0.80–0.94	< 0.001
**Age (per year)**	0.97	0.96–0.97	< 0.001
**Sex**			
Female	Ref		
Male	0.95	0.77–1.16	0.59
**Location of arrest**			
Not public	Ref		
Public	1.78	1.53–2.07	< 0.001
**Witness status**			
Unwitnessed	Ref		
Witnessed	3.01	2.57–3.54	< 0.001
**CPR status**			
No CPR	Ref		
Bystander CPR	1.28	1.17–1.48	< 0.001
**Chest compression rate**			
50–99	Ref		
100–120	1.58	1.30–1.94	< 0.001
> 120	0.97	0.65–1.44	0.86
**Initial cardiac rhythm**			
Nonshockable	Ref		
Shockable	6.53	5.47–7.80	< 0.001
**Epinephrine**			
No	Ref		
Yes	0.10	0.08–0.12	< 0.001
**Advanced airway**			
No	Ref		
Yes	0.59	0.49–0.70	< 0.001
**Hypothermia management**			
No	Ref		
Yes	4.94	3.76–6.48	< 0.001
Sex * hypothermia	1.61	1.18–2.19	0.003

AIC 161549.14

The predictor with the largest effect size was the initial shockable rhythm. The model indicated that patients with an initial shockable rhythm had 6.53 times greater odds of survival with good neurological function than those with unshockable rhythm, holding all other predictors constant (95% CI 5.47–7.80, *p* < 0.001). Two variables, insertion of an advanced airway and administration of epinephrine, were associated with poor neurological outcome. The model also revealed a significant interaction between sex and hypothermia. Males derived greater benefit from hypothermia management than females, holding all other predictors constant (sex*hypothermia 1.61, 95% CI 1.18–2.19, *p* = 0.003) (Table 6).

## Discussion

We identified the significant predictors of survival with good neurological function at hospital discharge in a cohort of patients with OHCA from multiple North American regions. Our adjusted analysis revealed the following variables had a positive impact on favorable neurological function: younger age, shorter duration of EMS arrival to the scene, arrest in a public location, witnessed arrest, bystander CPR, quality of CPR represented by 100–120 chest compressions per minute, initial shockable rhythm, and induction of hypothermia. Two variables had negative impacts on neurological function, insertion of an advanced airway, and administration of epinephrine. Our analysis showed that the crude rate of neurological intact survival was higher in males than females. Our results also revealed that males derived significantly greater benefit from hypothermia management than females.

With the exception of age, all of the significant predictive variables were event or treatment characteristics. Of note, many of these variables are modifiable, for example bystander CPR, induction of hypothermia, EMS arrival time, and insertion of an advanced airway. Initiatives and strategies that can improve treatment characteristics, such as strategies to shorten duration of EMS arrival to the scene and training more residents in basic life support bystander CPR, could improve survival with good neurological outcomes [3].

An initial shockable rhythm was the predictor with the largest effect size. This finding is consistent with the findings of previous studies that reported a positive association between an initial shockable rhythm and survival [8, 11, 33]. Additionally, our analyses showed that advanced airway management and administration of epinephrine were associated with decreased odds of favorable neurological outcome. The insertion of an advanced airway may interrupt chest compressions, which results in decreased blood flow to the brain. This is a plausible explanation of the negative impact of advanced airway management on neurological outcome. In line with our results, a RCT compared an epinephrine group with a placebo group to determine if administration of epinephrine during OHCA is effective. The trial found that the proportion of survivors with severe neurologic damage was higher in the epinephrine group than in the placebo group (31.0% vs. 17.8%) [34]. A possible explanation of the negative impact of epinephrine is perhaps the peripheral vasoconstriction effect of epinephrine as reported in a previous animal study [35]. However, other animal studies showed an increase of cerebral flow with bolus doses of epinephrine [36]. Our dataset had limited data on epinephrine bolus doses. It is worth mentioning that advanced airway and epinephrine are indicated for patients with prolonged resuscitation; perhaps those with unshockable rhythms. Therefore, in our study, confounding by indication cannot be disregarded.

Our adjusted analysis also showed that the odds of good neurological function among those who received a CCR between 100 and 120 compressions per minute was 1.6 times higher than that of patients who received 50–99 compressions per minutes. Similar finding was reported by Idris et al. [37]. Interestingly, our analysis showed that chest compressions delivered at a rate higher than 120 compressions per minutes was associated with poor neurological function. A plausible explanation for this is that compressions delivered too quickly (> 120/min) may not allow the chest to recoil during CPR, resulting in poor blood flow to the brain.

With regards to the effect of sex, the bivariate analysis showed that males had a significantly higher rate of neurologically intact survival. However, this advantage disappeared after adjustment. In the series of models produced by the multivariable analyses, sex remained statistically significant until we added hypothermia and initial rhythm. Only in the models that included initial shockable rhythm and/or hypothermia was the variable “sex” no longer statistically significant. This suggests that sex as a biological variable has no impact on neurological function once initial shockable rhythm and hypothermia management are taken into account. Nevertheless, it is important to note survival with good neurological outcome was lower in females.

Our unadjusted analysis demonstrated significant bivariate correlation between sex and initial rhythm. A similar correlation was shown in a recent North American study that reported females had a lower proportion of OHCA with an initial shockable rhythm compared to males [17]. Our analysis also showed significant bivariate correlation between sex and hypothermia. Females had a lower proportion of receiving hypothermia management compared to males (13.3% vs. 17.0%, *p* < 0.001). While an initial shockable rhythm and hypothermia management contribute significantly to favorable neurological outcome, the lower proportion of females with an initial shockable rhythm and receiving hypothermia thus contribute to the lower crude rate of neurological intact survival in females. It is possible that the initial unshockable rhythms in females may be related to delays in CPR [21]. It also may be related to differences in the etiology of cardiac arrest. Novel procedures that lead to early identification of OHCA in females may confer considerable benefits. The reason for sex differences in hypothermia management is not clear; it could be gender-related reasons, such as inequitable care, or sex-specific reasons, such as different requirements concerning hypothermia intervention.

To further examine the effect of sex on the outcome in relation to the baseline characteristics, we stratified the sample by sex and ran the analysis for each of the sex subgroups. We then compared the effect of predictors in each model. We found that hypothermia and initial rhythm had noticeably larger effect sizes in males than females, while the magnitude of the effects of the other predictors were similar. The marked difference in the effect size by sex, with much lower ORs in females than males, suggests possible interaction effects. Examination of possible interactions revealed that males derived significantly greater benefit from hypothermia management than females. These results suggest effect modification of hypothermia by sex. While the benefit of hypothermia for males was obvious, it could be due to other confounders related to pre-arrest status, arrest characteristics, or in-hospital treatments that influence the outcome, i.e., the higher effect size of hypothermia for males could be just a consequence that males had more favorable presenting initial rhythms, more witnessed arrests, or more post arrest percutaneous coronary interventions than females. In-depth investigation of the reasons for the observed differences in the effect sizes of hypothermia is required.

Of note, the proportion of survivors with favorable neurological outcome in the cohort who did not receive hypothermia intervention was low (3.3%). This indicates that selection bias might have been introduced: patients deemed to have poor neurological outcome may have been declined for hypothermia. Thus, no hypothermia was associated with poor neurological outcome, when really it is just a marker of patients having poor clinical status.

This study analyzed data from 47 clusters that participated in the ROC CCC trial. One strength of this study is that it applied multilevel analysis to account for clustering effect. Not accounting for clustering effect can produce inaccurate results, including incorrect estimates of the standard errors for the level-one predictors, and increases in the odds of finding a relationship when one does not exist, i.e., inflated type 1 error [28].

Our study has several limitations. First, the study analyzed data from multiple North American regions and the results may not be generalizable elsewhere in the world. Second, our study is vulnerable to unmeasured confounders. Data on some variables, such as comorbidities, year, and time of the event were incomplete in the dataset and, therefore, not included in the analysis. Fourth, the study focused on prehospital interventions. Data on other in-hospital treatment variables, such as cardiac interventions and revascularization, were not available in the dataset and therefore not included in the analyses. In addition to sex differences in OHCA characteristics, there may be some differences in post arrest care. Females less often presenting with shockable rhythms and less often receiving invasive coronary interventions [15, 21]. These are possible confounders that may explain the observed difference in the effects of hypothermia by sex.

## Conclusions

We found 10 predictors of neurological function, eight predictors of favorable neurological outcome, and two predictors of poor neurological outcome, after OHCA. The predictors of favorable neurological function include younger age, shorter duration of EMS arrival to the scene, arrest occurring in a public location, witnessed arrest, bystander CPR, chest compression rate of 100–120 compressions per minute, initial shockable rhythm, and induction of hypothermia. The predictors of poor neurological outcome include insertion of an advanced airway and administration of epinephrine.

Males with OHCA have higher crude rates of neurologically intact survival. However, the adjusted rate was not statistically significant. This suggests that sex as a biological variable has no independent effect on neurological function. The magnitude of the effect of the hypothermia on neurological outcome was higher in males than females, suggesting effect modifications of hypothermia by sex. The observed differences may be due to sex differences in OHCA characteristics, differences in post arrest care, or differences in other hidden confounders. In-depth confirmatory studies on the hypothermia effect size by sex are required.

## Data Availability

The data that support the findings of this study are available from [the National Heart, Lung, and Blood Institute (NHLBI), Biologic Specimen and Data Repository Information Coordinating Center (BioLINCC), but restrictions apply to the availability of these data, which were used under license for the current study, and so are not publicly available. Data are however available from the authors upon reasonable request and with permission of BioLINCC.
